# *In vivo* Differentiation of Human Monocytes

**DOI:** 10.3389/fimmu.2019.01907

**Published:** 2019-08-13

**Authors:** Alice Coillard, Elodie Segura

**Affiliations:** ^1^Institut Curie, PSL Research University, INSERM U932, Paris, France; ^2^Université Paris Descartes, Paris, France

**Keywords:** human, monocyte, macrophage, dendritic cell (DC), inflammation

## Abstract

Circulating monocytes can infiltrate mucosal or inflamed tissues where they differentiate into either macrophages or dendritic cells. This paradigm is supported by numerous studies conducted in mice and in different *in vitro* settings for human cells. Determining whether it holds true *in vivo* in humans is essential for the successful design of monocyte-targeting therapies. Despite limitations inherent to working with human samples, there is accumulating evidence of the existence of *in vivo-*generated monocyte-derived cells in humans. Here, we review recent studies showing the recruitment of human monocytes into tissues and their differentiation into macrophages or dendritic cells, in normal or pathological settings. We examine the methods available in human studies to demonstrate the monocytic origin of infiltrating cells. Finally, we review the functions of human monocyte-derived cells and how they might contribute to pathogeny.

## Introduction

Numerous studies in mice have shown that monocytes circulate in the blood and are recruited to mucosal tissues or inflammation sites, where they can differentiate into monocyte-derived macrophages (mo-Mac) or monocyte-derived dendritic cells (mo-DC) (1, 2). In models of inflammatory disorders, monocyte-derived cells have been shown to exert a deleterious role, in particular by fueling the inflammation and inducing tissue damage. Blocking their recruitment to inflamed tissues, using nanoparticules that induce apoptosis (3) or that contain si-RNA against CCR2 (4), reduces inflammation and improves the pathogeny in mouse models of colitis, peritonitis, and atherosclerosis. Monocytes have therefore emerged in the past few years as an attractive therapeutic target.

However, findings from mouse models do not always translate to humans due to genetic, physiological, and environmental differences. In particular, whether mice represent an appropriate model for analyzing inflammatory responses and chronic inflammatory diseases has been controversial (5–7). Despite inherent limitations, observations in humans are therefore essential to complement mouse studies to fully understand monocyte biology and the contribution of monocyte-derived cells to inflammatory disorders.

## Monocyte Life Cycle

Circulating monocytes are classified into three subsets based on the expression of the surface markers CD14 and CD16: “classical” CD14^high^CD16-monocytes (around 85% of monocytes), “intermediate” CD14+CD16+ monocytes (5–10%) and “non-classical” CD14-CD16^high^ (5–10%) monocytes. The life cycle and relationship of these subsets has been the subject of recent *in vivo* studies.

Several lines of investigation point to a linear differentiation relationship between monocyte subsets. Using *in vivo* labeling with a short pulse of 6,6-2H2-glucose in healthy volunteers, two studies have reported the sequential enrichment in the blood of labeled CD14^high^ monocytes, then CD14+CD16+ monocytes and finally CD16^high^ monocytes (8, 9). Similarly, following autologous stem cell transplantation, CD14^high^ monocytes reappeared first in the blood after 7 days, followed by CD14+CD16+ monocytes and then CD16^high^ monocytes after 10 days (10). Moreover, after *in vivo* endotoxin challenge in healthy volunteers, monocytes disappeared from the circulation within 2 h with CD14^high^ monocytes recovering after 4 h, then CD14+CD16+ monocytes and CD16^high^ monocytes after 24 h (9, 11). Mathematical modeling indicated that CD14^high^ monocytes have a short lifespan in the blood of 1–2 days, before differentiating into CD14+CD16+ monocytes or disappearing from the circulation (8, 9).

Consistent with this model, single-cell RNA-seq (scRNA-seq) analysis of blood monocytes showed that CD14+CD16+ monocytes are a heterogeneous population with mixed transcriptional profiles (12). In an independent scRNA-seq analysis, purified CD14^high^CD16- monocytes were shown to contain two subsets: one with a typical transcriptional profile of CD14^high^ monocytes and one with a profile closer to CD16^high^ monocytes, suggesting that part of the CD14^high^ monocytes are already en route to differentiate before up-regulation of CD16 (13).

Collectively, these observations support the notion that CD14^high^ monocytes represent the precursor population of both CD16^high^ monocytes in blood and monocyte-derived cells in tissues.

## Monocyte Recruitment Into Tissues

In mice, circulating monocytes leave the bloodstream to infiltrate mucosal tissues or inflamed sites, or to reside in the spleen. What is the evidence that the same scheme applies to humans?

Several studies have shown monocyte recruitment in the context of acute inflammation. In dialysis-induced bacterial peritonitis, CD14+ monocytes number was increased in the peritoneum 1 day after infection (14). In the acute inflammation model of skin blister, a high proportion of CD14+ cells was observed 24 h after blister formation, suggesting monocyte recruitment (15). Similarly, CD14+ cell number increased in the nasal mucosa 12 h after allergen challenge in a model of allergic rhinitis (16) and in the bronchoalveolar lavage 8 h following LPS inhalation (17). Furthermore, S100A8/9+ cells accumulated in the bronchial mucosa of patients who died from asthma attack as compared to non-atopic controls (16). Monocytes also infiltrate the heart following acute myocardial infarction, as shown by the increase in CD14+CD16- and CD14+CD16+ cells as compared to heart tissue from donors who died of other causes (18). This strong influx of monocytes correlated with a decrease in the proportion of CD14+ cells in the bone marrow and spleen, suggesting that the spleen could be a monocyte reservoir in humans. This is consistent with the presence of *bona fide* monocytes in human spleen (19).

Monocytes also infiltrate tissues in chronic inflammation. In inflammatory bowel diseases (IBD) such as Crohn's disease (CD) and ulcerative colitis (UC), an increased infiltration of monocytes was described in the colon. Following injection of radiolabelled monocytes, radioactive CD14+ cells were detected in the intestine of patients with intestinal inflammation (20) and in joints of patients suffering from rheumatoid arthritis (RA) (21). Moreover, CD14^high^CD11c^high^ monocytes were increased in the inflamed mucosa of CD patients as compared to control samples (22–24). During multiple sclerosis (MS), infiltrating monocytes were detected in MS lesions (25). CD14+ CCR2+ CD16- MerTK- cells were identified in the synovial fluid from gouty arthritis patients, suggesting recruitment of monocytes (26). In cancer, monocytes were detected in lung tumors (27) and breast tumors (28) using scRNA-seq analysis.

Monocyte recruitment in steady-state tissues has also been evidenced in a few studies. Extravascular monocytes have been observed in lung from organ donors (29). Monocyte-derived cells have been described in non-diseased intestine, liver and skin (see below).

To conclude, there is ample evidence that, similarly to mice, human monocytes are recruited at steady state in tissues and mucosa to replenish the niche, and in acute and chronic inflammation. In these different contexts, monocytes will further differentiate.

## Demonstrating the Monocytic Origin of Cells Isolated From Human Tissues

In mice, tracking monocyte fate from blood to tissues can be accomplished by adoptive transfer or genetic lineage tracing methods. These techniques are obviously not directly transposable to humans. When working with human samples, it is necessary to use alternative approaches to demonstrate the monocytic origin of tissue myeloid cells.

Phenotyping is the most widely used technique. It relies on the postulate that phenotypic markers expressed by blood monocytes will persist in tissues after their differentiation, allowing the identification of monocyte-derived cells. CD14, CCR2, and CX3CR1 are some of the most commonly used markers (Table 1). However, these molecules are not exclusive of monocyte-derived cells. CD14 is also expressed by tissue macrophages derived from embryonic precursors (40). CX3CR1 is highly expressed by microglia (1) and pre-DC (12). Finally, a population of pre-DC-derived CCR2+ DC has been described in mouse intestine (41).

**Table 1:** Surface markers commonly used to distinguish DC, macrophages, and monocytes.

**Surface markers**	**cDC1**	**cDC2**	**Resident macrophages**	**mo-Mac**	**mo-DC**	**CD14 + monocytes**
CCR2	–	–	–	+	+	+
CD11b	–	Tissue dependent	+	+	+	+
CD11c	+	+	+	+	+	+
CD14	–	–	Tissue dependent	+	+	++
CD141	++	+	low	low	+	–
CD16	–	–	Tissue dependent	+	–	–
CD163	–	–	Tissue dependent	+	–	–
CD172a (Sirpa)	–	+	+	+	+	+
CD1a	–	Tissue dependent	–	–	+	–
CD1b	–	+	–	–	+	–
CD1c	–	+	–	–	+	–
CD206	–	Tissue dependent	Tissue dependent	+	++	–
CD209	–	–	+	+	+	–
CD226	+	–	–	–	+	–
CD64	–	Tissue dependent	+	+	+	low
CD88	?	?	?	+	+	–
Clec9A	+	–	–	–	–	–
CX3CR1	–	Tissue dependent	Tissue dependent	+	+	+
FceRI	–	+	–	–	+	–
HLA-DR	+	+	+	+	+	+
MerTK	–	–	+	+	–	–
S100A8/A9	–	–	–	+	+	++
Main references	(30–32)	(30–33)	(30, 33–35)	(13, 14, 35–37)	(13, 14, 37–39)	(31)

Recently, S100A8/9 (calprotectin, an intracellular protein) has been proposed as a reliable marker for monocytes and monocyte-derived cells (34). S100A8/9 was detected both at the mRNA and protein levels in circulating monocytes (35, 42, 43). In the intestine, S100A8/9 expression in DC inversely correlated with Flt3 expression and S100A8/9 was highly expressed in short-lived myeloid cells, but not in long-lived macrophages (34). Collectively, this evidence suggests that S100A8/9 can be used as a marker for monocyte-derived cells.

Phenotyping is an easy way to characterize cellular identity. However, most markers are not specific to monocytes and monocyte-derived cells, and are tissue-dependent. Analyzing a combination of markers can increase the robustness of this approach.

Chimerism in the context of transplantation is another method to demonstrate a monocytic origin. This approach is based on the fact that following transplantation, cells from the recipient will repopulate the transplanted tissue while long-lived cells remain of donor origin, resulting in cellular chimerism. Resident macrophages derived from embryonic precursors are self-maintaining (1). Studying the replacement of tissue resident macrophages by monocyte-derived cells can be performed by analyzing markers of donor-recipient mismatch. For example, cells derived from the recipient's monocytes were evidenced in transplanted heart using *in situ* hybridization for Y chromosome (36). Cells derived from the donor or the recipient have also been distinguished by HLA-mismatch in the intestine (34, 35).

This method is an elegant way to analyse monocyte recruitment and differentiation in human tissues. However, it is not broadly available due to restricted access to transplanted tissue samples.

Another procedure to analyse monocyte fate is labeling, *ex vivo* or *in vivo*. For example, autologous monocytes were radiolabelled *ex vivo* and re-injected to patients with IBD (20) or RA (21). Monocytes can also be labeled *in vivo* through injection of ultra small particle iron oxide (USPIO). Labeled cells were observed in brain lesions of MS patients (25). This method allows a direct tracking of monocytes. However, it remains unconventional as it requires very specific procedures and ethics agreements.

Specific gene expression signatures can also be used to infer developmental origin. This approach is based on the idea that ontogeny will leave a transcriptomic imprint in monocyte-derived cells. One method is to perform comparative transcriptomic analysis, including blood monocytes as a reference. As an example, intestinal CD103-SIRPa+ cells (44) or CD14+ cells (34) clustered with blood monocytes, suggesting that these populations are related. Another method is to use transcriptional signatures specific of mo-Mac and mo-DC for enrichment analysis. scRNA-seq data of macrophages found in human fibrotic lung was annotated with signatures from bulk RNA-seq data from mouse macrophages and DC (45). This analysis revealed that pro-fibrotic macrophages may originate from monocytes. Similarly, scRNA-seq data from human ascite myeloid cells was analyzed using gene signatures generated from bulk RNA-seq of tissue-derived and *in vitro*-generated mo-DC and mo-Mac (46).

This approach has the advantage of being unbiased and requires no prior knowledge of putative “marker genes.” However, it is essential to use robust transcriptomic signatures as a reference.

The monocytic origin of tissue macrophages and DC can be demonstrated using various techniques. As none of these methods can lead to definitive conclusions, it is necessary to combine them to provide strong evidence that the cells of interest derive from monocytes.

## *In vivo* Differentiation Into MO-Mac and MO-DC

Human monocytes can be differentiated *in vitro* into mo-Mac or mo-DC in various culture conditions. However, do monocytes have the capacity to differentiate into both macrophages and DC *in vivo* in humans?

### How to Distinguish mo-Mac From mo-DC?

Distinguishing DC from macrophages by phenotyping is challenging as they share a lot of markers. MerTK, CD68, CD163, and the transcription factor MAFB are considered robust markers of macrophages, while DC express CD1a, CD1b, FcεRI, and CD226 (Table 1). Other techniques can help confirming cell identity. Analyzing cell morphology is a robust method for this (14, 36, 37, 47). Macrophages are large cells containing many phagocytic vesicles. By contrast, DC are smaller and display dendrites on their surface. Finally, transcriptomic approaches can also be used to distinguish mo-Mac from mo-DC, for instance by performing enrichment analysis for transcriptional signatures specific of DC and macrophages (34, 44, 46, 48–50).

### Identification of mo-Mac and mo-DC in Human Tissues

Many studies describe the presence of mo-Mac in different tissues at steady state. In the small intestine, two subsets of macrophages were replaced 3 weeks following transplantation by recipient cells (35), demonstrating that they derive from monocytes. Monocytes also participate in the replenishment of skin macrophages (33). Following allogeneic hematopoietic stem cell transplantation, CD14+ cells reappeared after 8 days in blood and after 12 days in normal skin. This sequential detection of CD14+ cells suggested that blood monocytes give rise to skin CD14+ macrophages (33). Monocyte differentiation into mo-Mac also occurs in tissues with lower self-renewal capacities. For example, mo-Mac were identified in the heart (36), lung (43), and liver (51).

Furthermore, mo-Mac have been described in different inflammatory settings. The increased presence of macrophages has been observed in the colon of IBD patients (52, 53). CCR2 expression on their surface suggested their monocytic origin. In the cantharidin-induced skin blister model, following monocyte recruitment, HLADR+CD14+CD16+ cells increase their expression of CD163 suggesting that they differentiate into mo-Mac (15). In dialysis-induced peritonitis, CCR2+ mo-Mac are increased in the peritoneal cavity as compared to normal dialysis (14). Finally, mo-Mac are detected in tumors. In glioma patients, mo-Mac were identified in the brain by CX3CR1 expression and transcriptomic analysis (49). In melanoma, scRNAseq analysis evidenced one population expressing both macrophage and monocyte genes suggesting the presence of mo-Mac (48).

Similar findings apply to mo-DC. At steady state, mo-DC are mainly described in the intestine. Intestinal SIRPa+CD103- DCs (44) and SIRPa+CD103-CD14+ DCs (34) are transcriptionally related to blood monocytes. Moreover, S100A8/9 expression suggested that this population derives from monocytes (34). The presence mo-DC expressing CCR2 and S100A8/9 has also been suggested in non-diseased lung (54). In CD, CD14+CD163-MerTK- cells from inflamed gut exhibited a typical DC morphology and scRNA-seq showed signatures of monocyte lineage, suggesting that these cells are mo-DC (47). CCR2+ DC were also evidenced in the peritoneum at steady-state (12).

There is also evidence of monocyte differentiation into mo-DC in an inflammatory context. In atopic dermatitis and psoriasis, early studies have suggested monocyte differentiation into mo-DC (55–57). An increased proportion of “inflammatory” DC was found in atopic dermatitis and psoriasis patients in comparison to healthy skin (55). Their phenotype (CD1a+ FceRI+ CD206+) is reminiscent of that of mo-DC identified in subsequent studies. Similarly, DC displaying a phenotype consistent with mo-DC were observed in pleural effusions of tuberculosis patients (58) and evidenced in colorectal and breast tumors (38, 50). In lung cancer, phenotypic analysis as well as transcriptomic signatures in scRNAseq data suggested the presence of mo-DC (27, 38, 59). Using gene signatures, peritoneal ascite DC from ovarian cancer patients were also identified as mo-DC (46).

Collectively, these observations relying on phenotypic, morphological and transcriptomic analysis support the notion that human monocytes can differentiate *in vivo* into both macrophages and DC.

## Functional Properties of MO-Mac and MO-DC

Classical DC and tissue resident macrophages play major roles in the initiation and resolution of immune responses. Do the DC and macrophages derived from monocytes display the same functions as their classical counterparts, or have specific functional properties?

### Secretion of Soluble Mediators

A major property of myeloid cells is the secretion of soluble mediators. Mo-DC and mo-Mac have been reported as strong producers of pro-inflammatory cytokines such as TNFα and IL1β. Mo-Mac from healthy intestine or from the colon of CD and UC patients secreted higher levels of TNFα as compared to their tissue resident counterparts, with or without *ex vivo* restimulation (35, 52, 60). Mo-DC from the inflamed colon of CD patients produced high levels of IL1β (36, 50). Peritoneal ascites mo-DC and mo-Mac also secrete high levels of TNFα and IL1β (37). Furthermore, heart mo-Mac are potent producers of IL1β in contrast to CCR2- tissue resident macrophages (36).

IL23 secretion is more specific to mo-DC. Mo-DC from the inflamed intestine of CD patients or from peritoneal ascites of cancer patients secreted significant levels of IL23 with (37) or without *ex vivo* restimulation (47). Similar results were obtained with mo-DC from pleural effusions of tuberculosis patients restimulated *ex vivo* with *Mycobacterium tuberculosis* (58). Although IL23 seems to be mainly produced by mo-DC, it has also been reported for mo-Mac from CD patients (52). By contrast, IL12 is produced by mo-DC but not mo-Mac (46).

Finally, a few studies reported the production of the anti-inflammatory cytokine IL-10 by mo-Mac. In the context of CD, mo-Mac from inflamed colon secreted high levels of IL-10 with (52) or without restimulation (60). *Il10* mRNA levels were upregulated in mo-Mac from CD patients after 24 h of culture without any stimulatory signal (36). Finally, mo-Mac from glioma were enriched for *Il10* expression (49).

Taken together, these findings indicate that mo-DC and mo-Mac are strong producers of pro-inflammatory cytokines, which are essential for the recruitment of immune cells at an injured site and for the initiation of immune responses. However, in chronic inflammation, cytokine secretion by mo-DC and mo-Mac is exacerbated and contributes to the pathogenesis.

### Fibrosis

Macrophages are key actors in wound healing and tissue repair by secreting growth factors for fibroblasts. The dysregulation of this mechanism can lead to fibrosis (61). There is evidence that mo-Mac participate in fibrosis. CX3CR1+ mo-Mac expressing pro-fibrotic Platelet Derived Growth Factor AA (PDGFAA) accumulated in fibrotic regions of lungs in comparison with non-fibrotic regions (45). Mo-Mac from cardiomyopathic heart expressed genes coding for growth factors and extracellular matrix components known to be involved in fibrosis (36). Moreover, these mo-Mac accumulated in scar or fibrotic tissues regions.

### CD8 T Cell Responses

Few studies have investigated the role of monocyte-derived cells in CD8 T cell responses. Both mo-DC and mo-Mac isolated from peritoneal ascites were able to cross-present antigens using a non-conventional intracellular pathway (46), consistent with another study using peritoneal mo-Mac and mo-DC from peritoneal dialysis (14). Of note, the ability to cross-present was specific of mo-Mac as compared to lymphoid organ macrophages (62). However, only mo-DC could provide costimulatory signals for the differentiation of effector cytotoxic CD8 T cells (46).

### CD4 T Cell Responses

One of the major roles of classical DC is to orient CD4 T cell polarization. Several studies have shown that mo-DC, but not mo-Mac, have the same property. Mo-DC isolated from synovial fluid of RA patients were better activators of CD4 T cell proliferation than mo-Mac from the same environment and induced Th17 polarization (37, 63). Th17 polarization was also induced by mo-DC from pleural effusions of tuberculosis patients (58) and from the inflamed colon of CD patients (47). Of note, mo-DC from synovial fluid of RA patients and from pleural effusions of tuberculosis patients were able to induce the proliferation of autologous CD4 T cells, showing that they can present antigens that were captured *in vivo* (58, 63). This Th17 polarization was associated with high secretion of IL-23 which is known to promote Th17 cells (37, 47, 58). In other studies, mo-DC from healthy small intestine or inflamed mucosa of CD patients preferentially induced Th1 polarization over Th17 (44, 52). This IFNγ production contributed to the pathogenesis of CD (52).

Finally, mo-DC from synovial fluid of RA patients and from peritoneal ascites induced CXCL13 secretion by CD4 T cells, suggesting Tfh polarization (64).

Collectively, these observations underline the capacity of mo-DC to polarize naïve CD4 T cells. In particular, Th17 polarization could contribute to the maintenance of chronic inflammation in Th17-driven pathologies such as RA and IBD.

In conclusion, mo-DC and mo-Mac share with their classical counterparts some of their hallmark functions (T cell stimulation for DC and tissue repair for macrophages). Ontogeny seems to influence mostly cytokine secretion, with mo-DC and mo-Mac being stronger producers of pro-inflammatory mediators than classical DC or resident macrophages from the same tissues. More studies using side-by-side comparisons will be needed to confirm this mixed functional profile.

## Conclusion

Despite methodological limitations inherent to human samples, numerous studies support the notion that human monocytes can differentiate *in vivo* into DC or macrophages. This process occurs at steady state to replenish the niche, but can also play a major role in the initiation and maintenance of chronic inflammatory diseases.

## Author Contributions

All authors listed have made a substantial, direct and intellectual contribution to the work, and approved it for publication.

### Conflict of Interest Statement

The authors declare that the research was conducted in the absence of any commercial or financial relationships that could be construed as a potential conflict of interest.
